# Vitamin D Status and Response to Supplementation as Predictive Factors for Early Remission in Polymyalgia Rheumatica: A Retrospective Longitudinal Investigation

**DOI:** 10.3390/nu17172839

**Published:** 2025-08-31

**Authors:** Elvis Hysa, Serena Balito, Giulia Davoli, Elisa Caratto, Giulia Bernardi, Emanuele Gotelli, Rosanna Campitiello, Carmen Pizzorni, Sabrina Paolino, Alberto Sulli, Vanessa Smith, Maurizio Cutolo

**Affiliations:** 1 Laboratory of Experimental Rheumatology, Academic Division of Clinical Rheumatology, Department of Internal Medicine, University of Genova, 16132 Genova, Italy; elvis.hysa@edu.unige.it (E.H.); sere94.balito@gmail.com (S.B.); giuliadavoli98@libero.it (G.D.); carattoelisa@gmail.com (E.C.); giulia.bernardi100@gmail.com (G.B.); emanuele.gotelli@unige.it (E.G.); campitiellorosanna@gmail.com (R.C.); carmen.pizzorni@unige.it (C.P.); sabrina.paolino@unige.it (S.P.); albertosulli@unige.it (A.S.); 2 Department of Experimental Medicine (DIMES), University of Genova, 16132 Genova, Italy; 3 IRCCS Ospedale Policlinico San Martino, 16132 Genova, Italy; 4 Department of Internal Medicine, Ghent University, 9000 Ghent, Belgium; vanessa.smith@ugent.be; 5 Department of Rheumatology, Ghent University Hospital, Corneel Heymanslaan 10, 9000 Ghent, Belgium; 6 Unit for Molecular Immunology and Inflammation, VIB Inflammation Research Center (IRC), 9052 Ghent, Belgium

**Keywords:** polymyalgia rheumatica, vitamin D, 25-hydroxyvitamin D, remission, glucocorticoids, disease activity

## Abstract

**Background/Objectives:** Polymyalgia rheumatica (PMR) is a relatively common inflammatory rheumatic disease of the elderly. The role of vitamin D remains unclear in this condition. The endpoints of this study were to assess 25-hydroxyvitamin D [25(OH)D] serum concentrations in PMR patients with active disease compared to elderly controls and to determine if baseline levels or changes following supplementation [delta 25-hydroxyvitamin D, Δ25(OH)D] were associated with improved clinical outcomes. **Methods:** In this retrospective, case–control study, 29 PMR patients (55% males, 75.24 ± 9.6 years old, disease duration of 3.8 ± 3 months) were included, meeting the 2012 EULAR/ACR classification criteria, with 29 age- and sex-matched controls without systemic inflammatory rheumatic diseases. We assessed demographic, clinical and laboratory features for PMR patients, including baseline 25(OH)D serum concentrations, disease activity (polymyalgia rheumatica activity score), and serum inflammatory biomarkers. A subgroup of them (*n* = 25) was followed longitudinally, for an average period of 21.1 ± 17.7 months, to evaluate the association between Δ25(OH)D and clinical outcomes at follow-up using multivariate logistic regression. **Results:** Although lower than the normal reference values, baseline 25(OH)D concentrations did not differ significantly between PMR patients and controls (21.6 ± 9.2 vs. 22.7 ± 11.3 ng/mL, *p* = 0.66) and did not predict long-term clinical outcomes. However, after only 3 months of supplementation, the increase in 25(OH)D concentrations was significantly associated with a remission status, and patients in remission showed a significant increase in 25(OH)D compared to those with persistent disease activity (+22.02 vs. +1.33 ng/mL, respectively; *p* = 0.044). Notably, in the multivariate model, this Δ25(OH)D was the strongest independent predictor of remission (OR = 2.89; 95% CI [1.60–4.11]), an effect independent of prednisone dosage prescribed at first visit (*p* = 0.32) and glucocorticoid exposure at third month (*p* = 0.12). **Conclusions:** Individual’s response of PMR patients to supplementation of vitamin D seems to be a robust independent predictor of early clinical remission achievement. Interestingly, optimizing vitamin D supplementation based on individual responsiveness may represent a valuable adjunctive strategy in PMR management.

## 1. Introduction

Polymyalgia rheumatica (PMR) is a relatively common inflammatory rheumatic disease predominantly affecting elderly adults, with peak incidence occurring between 70 and 75 years of age [1]. Characterized by pain and stiffness in the shoulders, neck, and hips, PMR significantly impairs patients’ quality of life [2]. In contrast to rheumatoid arthritis (RA), PMR does not cause structural joint damage even without glucocorticoid treatment [3]. However, in 16–23% of cases, PMR occurs concurrently with giant cell arteritis (GCA), a large-vessel vasculitis primarily affecting temporal arteries and the aorta [4]. Early recognition of large-vessel vasculitis in PMR patients is crucial, given the potential for severe vascular complications, including vision loss, stroke, and aortic aneurysms [5].

The immune pathophysiology of PMR is complex and multifaceted [6]. A recent systematic literature review positions PMR within a spectrum between autoinflammation and autoimmunity, with a predominant tendency toward autoinflammatory mechanisms characterized by macrophage activation and T helper 1 responses in the synovial tissue of affected bursae, notably without detectable pathogenic autoantibodies [7,8]. The triggers of PMR remain only partially understood, with hypotheses including viral or bacterial infections with neurotropic and respiratory tropism, unknown agents exhibiting seasonal variation, psychophysical stress, ultraviolet exposure, and exposure to certain drugs or vaccines (including COVID-19 and influenza vaccines) in genetically predisposed individuals [9,10,11,12,13].

Notably, PMR exhibits striking geographical variation in prevalence, being substantially more common in Northern European countries, with prevalences of 34–113 per 100,000 people, and in regions of the United States with Scandinavian ancestry, such as Minnesota [14,15]. This distribution pattern suggests contributions from both genetic and epigenetic factors, as well as potential environmental influences, including nutrients and sun exposure, which directly affect vitamin D synthesis through cutaneous photosynthesis and subsequent serum concentrations [9].

Numerous observational studies have documented associations between vitamin D serum concentrations and both the development of autoimmune or inflammatory rheumatic diseases and disease activity in established conditions, including RA, systemic lupus erythematosus, and systemic sclerosis [16,17]. Additionally, in vitro evidence suggests that vitamin D possesses immunomodulatory properties that may influence inflammatory processes [18,19].

Despite these broader associations, data specifically examining vitamin D status in PMR patients remain limited [20]. To our knowledge, only a single letter to the editor has reported insufficient vitamin D levels in eight patients with PMR and GCA, though this study lacked detailed clinical characterization and appropriate control groups, highlighting the need for a more comprehensive investigation of this relationship [21].

Therefore, the aim of this study was to assess 25-hydroxyvitamin D [25(OH)D] serum concentrations in consecutive PMR patients assessed at baseline in our Rheumatology Division and to compare the values with controls of similar age and sex without any systemic inflammatory rheumatic disease. Secondarily, we aimed to correlate 25(OH)D with clinical and laboratory parameters and assess whether baseline serum concentrations and the variations of 25(OH)D serum concentrations [Δ25(OH)D] evaluated longitudinally were associated with specific clinical outcomes in a subgroup of PMR patients.

## 2. Methods

### 2.1. Study Design and Participant Recruitment

This retrospective, observational, case–control study was conducted on patients recruited between January 2022 and June 2025. This study adhered to Good Clinical Practice principles and the Declaration of Helsinki. All participants provided informed consent for the retrospective utilization of anonymized clinical data for research purposes (MODHSMTD4_0003). This study was approved by the local Ethical Board Committee of the Ospedale Policlinico San Martino, Genova, Italy (EC 2023/4301).

The case group consisted of 29 patients diagnosed with PMR, all of whom fulfilled the 2012 European Alliance of Associations for Rheumatology (EULAR)/American College of Rheumatology (ACR) classification criteria [22]. To ensure the study focused on isolated PMR, exclusion criteria were applied. These included concurrent vitamin D supplementation; medical comorbidities known to affect vitamin D metabolism (e.g., chronic kidney disease, liver failure, malabsorption syndromes); and any concurrent signs or symptoms suggestive of GCA. Furthermore, patients who developed GCA or received an alternative diagnosis (e.g., elderly-onset rheumatoid arthritis, calcium pyrophosphate deposition disease) during the follow-up period were excluded from the final analysis (Supplementary Figure S1).

The control group comprised individuals matched for age and sex, who did not have systemic inflammatory rheumatic diseases. These participants were enrolled consecutively during the same study period and were subject to the same exclusion criteria regarding vitamin D supplementation and metabolic comorbidities.

### 2.2. Data Collection and Clinical Assessments

At baseline, a comprehensive dataset was collected for all participants. This included demographic information, anthropometric measurements (height, weight, body mass index [BMI]), detailed medical history, and current therapeutic regimens.

For the PMR cohort, the baseline clinical assessment systematically recorded disease duration, morning stiffness, constitutional symptoms (fever and weight loss), and GCA-related symptoms (headache, jaw claudication, visual disturbances). A detailed musculoskeletal examination documented pain in the shoulder and pelvic girdles and the spine, as well as prior use of glucocorticoids (GCs). Physical evaluation involved assessing for pain upon palpation of specific anatomical sites (long head of the biceps, subacromial area, ischiatic and trochanteric bursae, sacroiliac joints) and pain during active mobilization of the shoulder or pelvic girdles, with findings recorded dichotomously (present/absent). Additional assessments included upper limb elevation capacity, the presence of peripheral arthritis, and examination for temporal artery abnormalities, vascular bruits, and extremity claudication.

### 2.3. Outcome Measures and Disease Activity

Disease activity and patient-reported outcomes were evaluated using standardized measures. A 10 cm visual analog scale (VAS) was used to quantify pain, completed independently by both the patient and the physician. Functional capacity of the upper limbs was assessed using the upper limb elevation (EUL) score, graded on a 0–3 scale: 0 (normal elevation above the shoulder girdle), 1 (elevation up to the shoulder girdle), 2 (elevation below the shoulder girdle), and 3 (no elevation possible).

Overall disease activity in the PMR cohort was quantified using the polymyalgia rheumatica activity score (PMR-AS) [23]. This composite index incorporates five variables: C-reactive protein (CRP, mg/dL), patient global assessment (VAS, 0–10), physician global assessment (VAS, 0–10), duration of morning stiffness (minutes), and the EUL score. The PMR-AS was calculated using the formula: PMR-AS = CRP (mg/dL) + Patient VAS + Physician VAS + (0.1 × Morning Stiffness [min]) + EUL Score.

Disease activity was stratified based on the PMR-AS score: high activity (>17), medium activity (7−17), and low activity or remission (<7).

### 2.4. Follow-Up and Endpoint Definitions

Patients in the PMR cohort underwent follow-up evaluations at three-month intervals. Inclusion in the longitudinal analysis required a minimum follow-up duration of three months. At each visit, data were collected on the current daily and cumulative prednisone dosage, the initiation of any disease-modifying anti-rheumatic drugs (DMARDs), and laboratory markers, including the erythrocyte sedimentation rate (ESR), CRP, and serum 25(OH)D concentrations (post-supplementation).

A disease relapse was defined as the recurrence of PMR-related symptoms (aching and stiffness in the shoulder and pelvic girdles) in conjunction with elevated inflammatory markers (ESR or CRP) [24]. Remission was defined according to the consensus criteria by Dejaco et al., requiring all of the following: duration of morning stiffness < 15 min, ESR < 20 mm/h, CRP < 0.5 mg/dL, patient and physician global VAS < 10 mm, absence of pain on active and passive shoulder mobilization, normal upper limb elevation, and a stable or decreasing prednisone dosage [24].

### 2.5. Laboratory Tests

A baseline laboratory panel was performed for all participants. This included inflammatory biomarkers (erythrocyte sedimentation rate [ESR, mm/h] and C-reactive protein [CRP, mg/dL]) and a complete blood count (hemoglobin [Hb, g/dL], platelets [PLT, ×10^9^/L], white blood cells [WBC, ×10^9^/L]). Biochemical analysis assessed renal and liver function, alongside bone metabolism markers including serum calcium, phosphorus, parathyroid hormone (PTH), and 25(OH)D. To aid in differential diagnosis, rheumatoid factor (RF) and anti-citrullinated protein antibodies (ACPAs) were also measured. Finally, the season of blood withdrawal was recorded for all participants to account for potential seasonal variations in vitamin D levels.

### 2.6. Statistical Analysis

The normal distribution of metric data was verified before each statistical test, both analytically with the Kolmogorov–Smirnov test and graphically with Q-Q plots. In the case of normal distribution, parametric tests, such as the independent Student t-test, were used. Data non-symmetrically distributed were analyzed with non-parametric tests, such as Mann–Whitney, Kruskall–Wallis, or Friedman. Categorical variables, such as frequencies, were compared with the Chi-squared test. Correlations between variables were calculated using Pearson’s correlation for normally distributed data or Spearman’s rank correlation for non-normally distributed data.

Potential confounding factors influencing baseline 25(OH)D levels in PMR patients were identified and analyzed. A stepwise approach was employed for variable selection: clinically relevant variables potentially affecting vitamin D status were initially included in univariate analysis, including age, sex, disease duration, prednisone intake, cumulative prednisone dosage until V0, BMI, and season of blood withdrawal (spring/summer vs. autumn/winter). Variables with *p* < 0.1 in the univariate analysis were subsequently entered into the multivariate model. This exploratory *p*-value threshold (*p* < 0.1) was chosen for the initial variable screening to avoid the premature exclusion of potential confounders that might be significant only in the context of the multivariate model. Multiple linear regression was performed when the dependent variables were continuous, whereas logistic regression was utilized when the dependent variables were categorical.

For longitudinal analysis, changes in 25(OH)D serum concentrations from baseline (delta values) were calculated. The Mann–Whitney U test was used to compare the change in 25(OH)D concentrations at three and six months between patients who achieved remission and those with persistent disease activity. Multivariate logistic regression models were constructed to assess the predictive value of baseline and delta 25(OH)D for clinical outcomes, such as remission, adjusting for potential confounders, including the dosage of baseline prednisone and the cumulative prednisone exposure at each visit. All analyses were performed with a significance level set at *p* < 0.05. Statistical analysis was performed with Datatab^®^ (https://datatab.net/, months of access: June and July 2025). Figures were generated using the Matplotlib library (https://matplotlib.org/, months of access: June and July 2025)

## 3. Results

### 3.1. Descriptive Results Related to the Included PMR Population

Sixteen out of twenty-nine (55%) PMR patients were male with a mean age of 75.24 ± 9.6 years, a BMI of 26 ± 3.6, and a disease duration of 3.8 ± 1 months. Twenty out of twenty-nine patients (69%) were already treated with GCs for a median duration of 30 (13–106) days and a mean cumulative prednisone dosage of 258 (88–458) mg of prednisone equivalent. The mean PMR-AS at first visit was 17.78 ± 6.11; in particular, 15 patients (52%) were in high disease activity, 13 patients were in moderate disease activity (45%), and 1 patient (3%) was in low disease activity. The major comorbidities of the patients were systemic arterial hypertension (83%) and dyslipidemia (55%). Two patients showed positivity for rheumatoid factor (RF) IgM at low titres (<40 IU/mL), whereas the other patients had a negative autoantibody profile. The detailed clinical and laboratory features of PMR patients at baseline are reported in Table 1.

### 3.2. Descriptive Results Related to the Included Control Population

The comparative population consisted of 29 age- and sex-matched individuals mainly diagnosed with osteoarthritis (mainly in the hands, 69%), serving as a non-systemic inflammatory disease control group. Their demographic profile was nearly identical to the PMR cohort, with a mean age of 75.0 ± 9.1 years and 55.2% being male. The prevalence of major comorbidities, such as systemic arterial hypertension (62%) and dyslipidemia (55%), was also comparable. In contrast to the PMR group, all controls were glucocorticoid-naïve and, as expected, had normal baseline inflammatory markers (median ESR of 17.50 mm/h; mean CRP of 3.49 mg/L). The detailed characteristics of age- and sex-matched elderly controls are reported in Supplementary Table S1.

### 3.3. Comparison of 25OHD Serum Concentrations Between PMR Patients and Controls

Serum 25(OH)D concentrations were found to be lower than normal reference values but did not differ significantly between PMR patients and controls (21.6 ± 9.2 vs. 22.7 ± 11.3 ng/mL, *p* = 0.66). This was observed even when comparing the values of GC-naïve PMR patients vs. controls (30.1 ± 20.1 vs. 22.7 ± 11.3 ng/mL, *p* = 0.16). Additionally, no significant differences were observed in the other parameters of the osteometabolic profile between the whole cohort of PMR patients vs. controls (Table 2).

### 3.4. Correlations Between 25(OH)D Serum Concentrations and Clinical and Laboratory Features in PMR Patients

An analysis of individual clinical and laboratory parameters did not reveal any statistically significant correlations with serum 25(OH)D concentrations except for a non-significant inverse trend between 25(OH)D and CRP serum concentrations (r = −0.36, *p* = 0.063, Table 3). Similarly, baseline 25(OH)D concentrations did not differ significantly between GC-treated and treatment-naive PMR patients (*p* = 0.10). Interestingly, in a multivariate model, both increasing age (β = −0.43, *p* = 0.02) and baseline prednisone intake (β = −0.5, *p* = 0.01) were identified as significant independent factors negatively associated with 25(OH)D serum concentrations (Table 4).

### 3.5. Outcomes of PMR Patients and Role of Baseline and Longitudinal Vitamin 25(OH)D Serum Concentrations on Clinical Outcomes

The clinical outcomes for a subgroup of 25 patients were assessed over a mean of 21.1 ± 17.7 months. This cohort, with a mean baseline 25(OH)D concentration of 20.7 ± 9.7 ng/mL and an initial prednisone dose of 15.0 ± 7.7 mg daily, experienced an average of 0.9 ± 1.3 relapses. The variability in the initial prednisone dosage reflects the real-life nature of the study; while clinicians followed international recommendations suggesting 12.5–25 mg/day, dosages were often tailored based on individual clinical conditions such as higher body mass index, severity of inflammatory markers, worse patient-reported outcomes, or comorbidities [25,26,27]. At the conclusion of the follow-up period, the prednisone discontinuation rate was 20%, and the requirement for DMARD therapy was 36% (Supplementary Figure S2). More specifically, eight patients received methotrexate, one received leflunomide, and one received tocilizumab.

**Figure 1:**
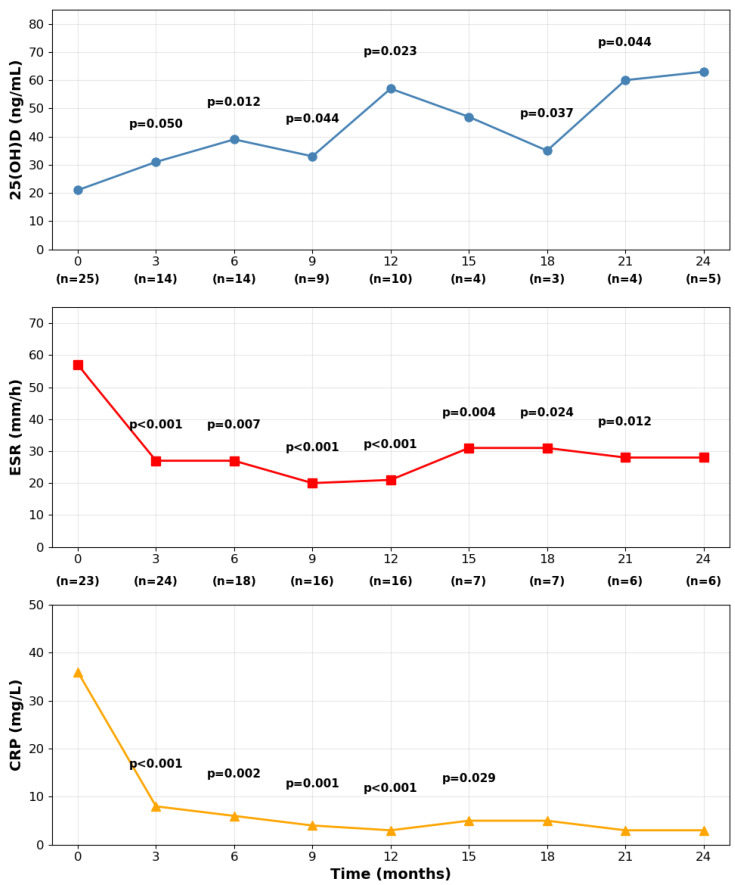
Longitudinal laboratory parameters in PMR patients.

Vitamin D supplementation was prescribed at the first visit (V0) to all patients. The majority received cholecalciferol (*n* = 22, 88%), with mean daily dosages of 2104.5 ± 743.7 international units (IU). Cholecalciferol was administered using different schemes: a monthly dosage of 25.000 IU (*n* = 12), weekly or every other week doses (*n* = 6), or a daily regimen (*n* = 4). Calcidiol was prescribed to three patients (12%) with a mean daily dosage of 1733.3 ± 230.9 IU and as daily drops. This initial supplementation protocol was then adjusted during subsequent follow-ups based on the observed changes in serum 25(OH)D levels, with the clinical goal of reaching and maintaining minimal sufficient concentrations (>40 ng/mL). Over the 24-month observation period, this tailored approach led to a statistically significant increase in serum vitamin D concentrations from baseline, which occurred concurrently with a significant and sustained reduction in inflammatory biomarkers, including both ESR and CRP (Figure 1).

The figure illustrates the significant changes in laboratory markers over a 24-month period, showing a progressive increase in serum 25(OH)D concentrations alongside a concurrent and sustained reduction in inflammatory markers (ESR and CRP) compared to baseline.

### 3.6. Influence of Baseline 25(OH)D Serum Concentrations on the Final Outcomes

To determine if vitamin D status at presentation influenced the long-term disease course, baseline 25(OH)D concentrations were assessed as a predictor for several clinical outcomes. The analysis found no statistically significant associations. Specifically, baseline vitamin D concentrations did not predict the total number of relapses (*p* = 0.214), the cumulative prednisone dosage (*p* = 0.320), the likelihood of successful prednisone discontinuation (*p* = 0.38), or the subsequent requirement for DMARD therapy (*p* = 0.946). These findings remained non-significant even after adjusting for the baseline prednisone dose (Supplementary Table S2).

### 3.7. Association Between Serum 25(OH)D Changes and Clinical Outcomes

**Figure 2:**
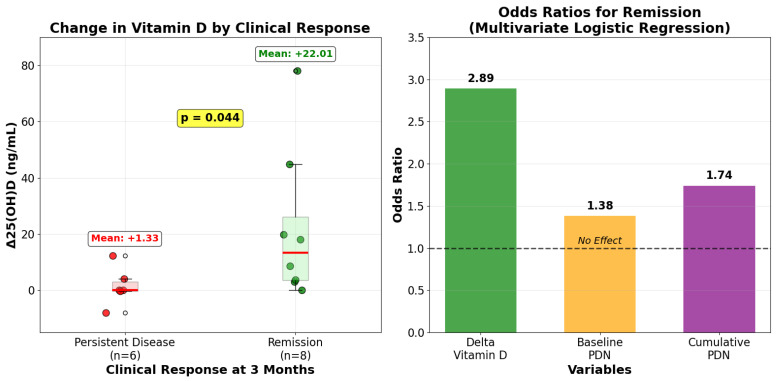
25-Hydroxyvitamin D changes as a predictor of clinical remission at three months in PMR.

At the three-month follow-up, the change in serum 25(OH)D [Δ25(OH)D] was significantly associated with clinical response. Patients who achieved remission (*n* = 8) exhibited a substantially greater increase in 25(OH)D concentrations (mean: +22.02 ng/mL) compared to those with persistent disease activity (*n* = 6; mean increase: +1.33 ng/mL; *p* = 0.044, Figure 2).

Notably, this finding was not attributable to baseline confounders, as the two subgroups were well-matched for key demographic, clinical, and laboratory characteristics (Supplementary Table S3).

Additionally, in the multivariate logistic regression model, the Δ25(OH)D from baseline to three months was identified as a statistically significant predictor of clinical remission. It demonstrated the highest odds ratio among all tested variables (OR = 2.89), with a 95% confidence interval of 1.60 to 4.11 (*p* < 0.05). In the same model, neither baseline prednisone dosage (OR = 1.38; 95% CI [0.29, 6.62]; *p* = 0.32) nor cumulative GC exposure (OR = 1.74; 95% CI [0.36, 8.35]; *p* = 0.12) was found to be a significant predictor of the remission status at the third month.

This differential response occurred despite the comparable daily vitamin D supplementation regimens between the groups (1852.8 ± 985.73 IU versus 1264.17 ± 804.68 IU, respectively; *p* = 0.21), although it did not correlate with changes in inflammatory markers such as ESR (*p* = 0.567) or CRP (*p* = 0.937).

An interesting reversal of this pattern was observed at the six-month follow-up. Patients with persistent disease activity showed a numerically larger, though not statistically significant, increase in 25(OH)D compared to those in remission (mean: 24.7 ± 27.6 ng/mL, *n* = 7 vs. mean: 8.3 ± 6.6 ng/mL, *n* = 7, *p* = 0.123), a finding with a large effect size (Cohen’s d = 0.82).

This might likely reflect confounding by indication, wherein clinicians intensified supplementation in patients with a suboptimal response. Due to progressive sample size reduction, further analyses of vitamin D dynamics were not performed beyond this timepoint.

The left panel displays a box plot comparing the change in serum 25(OH)D levels (delta vitamin D) at three months between patients who achieved remission and those who did not, showing a significantly greater increase in the remission group. The right panel presents the odds ratios from the multivariate logistic regression model, demonstrating that the change in vitamin D is the strongest predictor of achieving remission when compared to baseline and cumulative prednisone (PDN) exposure.

## 4. Discussion

This study represents the first comprehensive assessment of 25-hydroxyvitamin D [25(OH)D] status in PMR patients with detailed clinical correlations. Our observational analysis revealed no significant differences for the low serum vitamin D concentrations between PMR patients and age- and sex-matched elderly controls. This finding seems to suggest that vitamin D insufficiency may not constitute a primary pathogenic risk factor in PMR development, particularly given that both patient groups indeed demonstrated inadequately low vitamin D serum concentrations.

This finding on pathogenesis is nuanced by large-scale prevention trials. For instance, the VITAL study found that vitamin D supplementation significantly reduced the incidence of autoimmune disease overall (HR 0.78, 95% CI 0.61 to 0.99), while the favorable trend for PMR specifically did not reach statistical significance (HR 0.70, 95% CI 0.44 to 1.12) [28].

Interestingly, a trend toward inverse correlation between serum 25(OH)D concentrations and CRP levels was observed in the PMR cohort, supporting the possibility that vitamin D may influence inflammatory pathways. This relationship aligns with established findings in other autoimmune and inflammatory rheumatic conditions [29]. However, this was not statistically significant, which is further supported by the absence of a significant association between 25(OH)D concentrations and PMR activity score (PMR-AS), and further studies with larger cohorts are needed to establish if the correlation is significant, preferably in GC-naive patients.

An analysis of factors influencing baseline serum vitamin D concentrations identified age and concurrent prednisone treatment as significant negative predictors. These findings suggest that the most elderly PMR patients may require vitamin D supplementation, especially given the universal prescription of prednisone in PMR management [30]. Interestingly, despite well-documented seasonal variation in both vitamin D levels and autoimmune disease onset/severity [31], we did not observe seasonal effects on 25(OH)D concentrations at the time of blood sampling, likely attributable to our limited sample size.

The longitudinal component of our study showed the most interesting clinical results in vitamin D response that may have implications for PMR management. Most notably, the magnitude of vitamin D increase following supplementation emerged as a robust independent predictor of clinical remission at three months, demonstrating the highest odds ratio (OR = 2.89) among all examined variables in multivariate analysis. This association maintained statistical significance even after controlling for both baseline and cumulative prednisone exposure, suggesting that vitamin D responsiveness might operate through mechanisms independent of GC administration.

Specifically, the final active metabolite of vitamin D (being a secosteroid D hormone), namely the calcitriol (1,25(OH)D3), is known to inhibit the pro-inflammatory T helper 1 (Th1) responses and downregulate M1 macrophage polarization, both of which are considered key drivers in the autoinflammatory pathophysiology of PMR. By promoting a shift towards regulatory T-cell phenotypes and anti-inflammatory M2 macrophages, vitamin D could directly dampen the specific cellular pathways driving the disease [32].

In addition, since this effect was independent of initial GC therapy, our findings suggest that achieving vitamin D sufficiency may contribute to the clinical conditions that permit an earlier tapering of prednisone, particularly useful in elderly patients [33,34,35].

A primary limitation of this study is its small sample size (*n* = 29), which constrained the statistical power to detect more subtle associations. This was particularly relevant for assessing the correlation between 25(OH)D and markers of disease activity, as well as for analyzing seasonal variations and performing further subgroup analyses over time. The retrospective design inherently constrains causal inference, and the monocentric nature of this study, compounded by strict selection criteria that slowed recruitment by excluding patients without baseline 25(OH)D levels or those with prior supplementation, may limit generalizability to broader PMR populations. In addition, the study cohort was exclusively Caucasian, which may limit the applicability of our findings to other ethnic groups with different genetic backgrounds and vitamin D metabolism. Moreover, our baseline data reflects the first rheumatological visit rather than the initial onset of symptoms, an intrinsic constraint of the retrospective data collection.

Furthermore, the inclusion of patients already receiving GC therapy at baseline (69% of the cohort) represents a potential confounding factor, as GCs are known to influence vitamin D metabolism and bone homeostasis [36]. However, the high baseline inflammatory markers in these pre-treated patients simply reflect that their initial therapy was insufficient to control the active disease, which was a prerequisite for their inclusion in this real-world cohort. Similarly, the longitudinal ESR values should be interpreted in the context of our elderly cohort, for whom levels up to 30 mm/h can be within the normal range and are subject to inter-laboratory variability. While our multivariate analysis identified prednisone intake as a negative predictor of 25(OH)D levels, the lack of standardized dosing protocols and variable treatment durations may have introduced additional variability. Finally, we did not assess other potential confounders such as dietary vitamin D intake, sun exposure habits, skin phototype, or the use of sunscreen, which could significantly influence serum concentrations.

The lack of standardized vitamin D supplementation protocols may have introduced variability in treatment responses, though this limitation actually strengthens our findings by demonstrating the robustness of the vitamin D–remission association across different supplementation approaches.

Moreover, the exclusion of patients with concurrent GCA symptoms, while ensuring PMR disease homogeneity, limits the applicability of our findings to the broader spectrum of patients with PMR-GCA overlap syndromes.

Finally, the absence of data on pro-inflammatory cytokine serum concentrations precluded an investigation into the potential biological mechanisms, such as the immunomodulatory effects of vitamin D, that might have further explained the clinical benefits observed [37].

Further work warrants investigation to advance the understanding of vitamin D’s role in PMR pathophysiology and management. Prospective longitudinal studies with larger cohorts are ongoing to establish the potential existence of temporal relationships between vitamin D status and disease onset, activity, and treatment response in PMR. Such studies will incorporate a standardized assessment of potential confounders, including detailed dietary histories, sun exposure patterns, and comprehensive medication reviews. Interventional studies examining the efficacy of vitamin D supplementation in PMR patients should provide valuable insights into potential therapeutic benefits.

## 5. Conclusions

The present study provides the first comprehensive evaluation of vitamin D status in PMR, revealing that while low baseline 25-hydroxyvitamin D levels do not differ from age- and sex-matched elderly controls, individual vitamin D responsiveness to supplementation emerges as a significant independent predictor of clinical remission in early stages of the disease (from the third month).

The magnitude of vitamin D increase following supplementation demonstrated the highest odds ratio for remission among all examined variables, maintaining significance even after controlling for GC exposure.

These findings suggest that vitamin D optimization strategies based on individual responsiveness may represent a valuable adjunctive approach in PMR management in order to achieve the earliest remission.

## Figures and Tables

**Table 1:** Baseline clinical, demographic, and laboratory characteristics of PMR patients.

Parameters	Observed Values in PMR Patients (*n* = 29)
**Demographic Features**	
Male sex (*n*, %)	16/29 (55%)
Age (years), mean ± SD	75.24 ± 9.6
Disease duration (days), mean ± SD	113 ± 89
BMI, mean ± SD	26 ± 3.6
**Clinical features at first rheumatological visit** (*n*, %)	
Inflammatory pain in shoulder girdle	29 (100%)
Inflammatory pain in pelvic girdle	22 (76%)
Inflammatory pain in cervical spine	7 (24%)
Number of patients with prolonged morning stiffness	24 (83%)
Number of patients with peripheral arthritis	5 (17%)
New-onset headache ^#^	3 (10%)
Jaw claudication	0 (0%)
Number of patients with weight loss	3 (10%)
Number of patients with fever	5 (17%)
Lower limb claudication	0 (0%)
New-onset visual impairment	1 (3%)
**Comorbidities** (*n*, %)	
Systemic arterial hypertension	24 (83%)
Dyslipidemia	16 (55%)
Gastroesophageal reflux disease	7 (24%)
Type 2 diabetes mellitus	5 (17%)
Osteoporosis	5 (17%)
Previous history of arterial or venous thrombosis/acute cardiovascular event	5 (17%)
Benign prostatic hyperplasia	4 (14%)
Chronic atrial fibrillation	3 (10%)
**Current treatment** (*n*, %)	
Patients taking GCs at V0 (*n*, %)	20 (69%)
Median daily dosage of prednisone in GC-treated patients (mg), median (IQR)	5 (0–10)
Time of consumption of GCs for GC-treated patients (days), median (IQR)	30 (13–106)
Patients taking antihypertensive medications	24 (83%)
Statins	16 (55%)
PPIs	14 (48%)
Diabetes medications	3 (10%)
Analgesics (i.e., NSAIDs, paracetamol, tramadol)	7 (24%)
Supplements/vitamins (i.e., iron, folic acid, omega-3, carnitine)	5 (17%)
Urological medications	4 (14%)
Anticoagulants (i.e., warfarin or DOAC)	4 (14%)
Antiplatelets	4 (14%)
**Disease activity**	
PMR-AS mean ± SD	17.78 ± 6.11
Low disease activity (PMR-AS between 1.6 and 6.9), (*n*, %)	1 (3%)
Moderate disease activity (PMR-AS between 7 and 17), (*n*, %)	13 (45%)
High disease activity (PMR-AS > 17), (*n*, %)	15 (52%)
**Laboratory parameters**	
ESR (mm/h), mean ± SD	65 ± 41
CRP (mg/L), median (IQR)	71 (21–444)
WBC (10^9^/L), mean ± SD	9 ± 4
PLT (10^9^/L), mean ± SD	338 ± 100
RF positivity (*n*, %)	2 (7%)
ACPA positivity (*n*, %)	0 (0%)
25(OH)D (ng/mL), mean ± SD	22 ± 14
PTH (pg/mL), mean ± SD	47 ± 30
Ca (mg/dL), mean ± SD	9.5 ± 0.5
P (mg/dL), mean ± SD	3.7 ± 1

Legend with abbreviations: 25(OH)D: 25-hydroxyvitamin D; ACPA: anti-citrullinated protein antibodies; BMI: body mass index; Ca: calcium; CRP: C-reactive protein; DOAC: direct oral anticoagulant; ESR: erythrocyte sedimentation rate; EUL: elevation of the upper limbs; GCs: glucocorticoids; IQR: interquartile range; NSAIDs: nonsteroidal anti-inflammatory drugs; P: phosphorus; PLT: platelets; PMR-AS: polymyalgia rheumatica activity score; PPIs: proton pump inhibitors; PTH: parathyroid hormone; RF: rheumatoid factor; SD: standard deviation; V0: visit at time 0. ^#^ for patients reporting new-onset headache, GCA was ruled out clinically and, when necessary, with the vascular ultrasound of temporal and axillary arteries. In the table, words in bold represent the categories of descriptive characteristics for the cohort.

**Table 2:** Osteometabolic profile in PMR patients vs. controls.

Laboratory Parameters Related to Bone Profile	PMR Patients (*n* = 29)	Controls (*n* = 29)	*p*-Value
25(OH)D	21.57 ± 9.24	22.68 ± 11.28	0.66
PTH	52.01 ± 29.84	69.62 ± 38.09	0.07
Ca	9.49 ± 0.47	9.34 ± 0.30	0.07
P	3.27 ± 0.56	3.17 ± 0.96	0.19

Legend with abbreviations. 25(OH)D: 25-hydroxyvitamin D; Ca: calcium; P: phosphorus; PTH: parathyroid hormone.

**Table 3:** Associations and correlations between serum vitamin D concentrations and the clinical/laboratory characteristics of patients with PMR.

Comparisons of 25(OH)D Values in Patient Subgroups Based on Clinical Characteristics	
Patients with inflammatory pain in the hip vs. patients without	20.7 ± 9.5 vs. 24.4 ± 8.5, *p* = 0.5
Patients with inflammatory pain in the cervical column vs. patients without	20.7 ± 7.5 vs. 21.8 ± 9.9, *p* = 0.5
Patients with peripheral arthritis vs. patients without	24.0 ± 9.1 vs. 21.1 ± 9.4, *p* = 0.5
Patients with fever vs. patients without	15.5 ± 8.3 vs. 22.8 ± 9.1, *p* = 0.2
Patients with weight loss vs. patients without	19.9 ± 5.7 vs. 21.8 ± 9.6, *p* = 0.74
**Correlations between 25(OH)D concentrations and continuous clinical and laboratory variables**	
Duration of morning stiffness	r = −0.09, *p* = 0.63
VAS patient	r = −0.27, *p* = 0.16
VAS physician	r = −0.19, *p* = 0.34
PMR-AS	r = −0.1, *p* = 0.66
ESR	r = 0.07, *p* = 0.72
CRP	r = −0.36, *p* = 0.063 *
WBC	r = 0.11, *p* = 0.6
PLT	r = −0.25, *p* = 0.18

Legend with abbreviations: 25(OH)D: 25-hydroxyvitamin D; ACPA: anti-citrullinated protein antibodies; BMI: body mass index; Ca: calcium; CRP: C-reactive protein; DOAC: direct oral anticoagulant; ESR: erythrocyte sedimentation rate; EUL: elevation of the upper limbs; GCs: glucocorticoids; IQR: interquartile range; NSAIDs: nonsteroidal anti-inflammatory drugs; P: phosphorus; PLT: platelets; PMR-AS: polymyalgia rheumatica activity score; PPIs: proton pump inhibitors; PTH: parathyroid hormone; r: correlation coefficient; RF: rheumatoid factor; SD: standard deviation; V0: visit at time 0; VAS: visual analog scale; WBC: white blood cell count: * non-significant inverse trend.

**Table 4:** Univariate and multivariate analyses with predictors influencing 25-OH vitamin D serum concentrations in PMR patients. Independent, dependent variables and significant *p*-values (<0.05) are reported in bold.

Univariate Analysis	
Independent variables	Dependent variable **Serum 25(OH)D**
**Age**	β = −0.34, *p* = 0.07
**Sex**	β = −0.16, *p* = 0.39
**Disease duration**	β = 0, *p* = 0.98
**Prednisone intake**	β = −0.31, *p* = 0.1
**Cumulative prednisone dosage until V0**	β = −0.19, *p* = 0.35
**BMI**	β = 0.712, *p* = 0.1
**Season of blood withdrawal (spring/summer vs. autumn/winter)**	β = −0.33, *p* = 0.08
**Multivariate analysis**	
Independent variables	Dependent variable **Serum 25(OH)D**
**Age**	β = −0.43, ***p* = 0.03**
**Prednisone intake**	β = −0.49, ***p* = 0.02**
**BMI**	β = −0.34, *p* = 0.09
**Season of blood withdrawal (spring/summer vs. autumn/winter)**	β = −0.03, *p* = 0.87

## Data Availability

Data are available on reasonable request due to privacy reasons.

## Supplementary Materials

The following supporting information can be downloaded at: https://www.mdpi.com/article/10.3390/nu17172839/s1. Table S1. Baseline clinical, demographic, and laboratory characteristics of controls with non-inflammatory rheumatic disease; Table S2. Influence of baseline serum 25(OH)D concentrations on long-term clinical outcomes; Table S3. Differences in baseline features of patients achieving remission or persistent active disease at third month with available dosages of 25-hydroxy vitamin D serum concentrations at baseline and at third month; Figure S1. Flow diagram of participant selection; Figure S2. Clinical outcomes in PMR patients.
