# Supplementary material for: Vitamin D Status and Response to Supplementation as Predictive Factors for Early Remission in Polymyalgia Rheumatica: A Retrospective Longitudinal Investigation
Source: Nutrients. 2025 Aug 31;17(17):2839. doi: 10.3390/nu17172839 (PMC12430293; doi:10.3390/nu17172839)
Supplement: Supplementary file 1 [file nutrients-17-02839-s001.zip › nutrients-3836808-supplementary.pdf]

Parameter	Observed values
**Demographic features**	
Male sex (n, %)	16/29 (55.2%)
Age (years), mean ± SD	75.0 ± 9.1
BMI, mean ± SD	26 ± 3.6
**Clinical features at first rheumatological visit**	
Pain in the hands with mechanical features*, (n, %)	20/29 (69%)
Knee pain with mechanical features (n, %)	11/29 (38%)
Shoulder pain with mechanical features (n, %)	2/29 (7%)
Pelvic girdle pain with mechanical features (n, %)	1/29 (3%)
Pain in the cervical spine with mechanical features (n, %)	1/29 (3%)
Number of patients with peripheral arthritis (n, %)	0/29 (10%)
New-onset headache (n, %)	0/29 (0%)
Jaw claudication (n, %)	0/29 (0%)
Number of patients with weight loss (n, %)	2/29 (7%)
Number of patients with fever (n, %)	0/29 (0%)
Lower limb claudication (n, %)	0/29 (0%)
New-onset visual impairment (n, %)	0/29 (0%)
**Comorbidities (controlled by treatment)**	
Systemic arterial hypertension (n, %)	18/29 (62%)
Dyslipidemia (n, %)	16/29 (55%)
Gastroesophageal reflux disease (n,%)	5/29 (17%)
Type 2 diabetes mellitus (n, %)	5/29 (17%)
Osteoporosis (n, %)	3/29 (10%)
Previous history of arterial or venous thrombosis/acute cardiovascular event	4/29 /14%)
Chronic atrial fibrillation (n, %)	3/29 (10%)
**Current treatment**	
Glucocorticoids at V0 (n, %)	0/29 (0%)
Antihypertensive medications, n (%)	18/29 (62%)
PPIs, n (%)	8/29 (28%)
Diabetes medications, n (%)	3/29 (10%)
Analgesics (i.e. NSAIDs, paracetamol, tramadol) , n (%)	5/29 (17%)
Antiplatelets, n (%)	4/29 (14%)
Anticoagulants (i.e. warfarin or DOAC), n (%)	3/29 (10%)
Statins, n (%)	4/29 (14%)
**Laboratory parameters**	
ESR (mm/h), median (IQR)	17.50 (5.75-35.75)
CRP (mg/L), mean ± SD	3.49 ± 2.49
WBC (10^9/L), mean ± SD	5.83 ± 1.41
PLT (10^9/L), mean ± SD	206.96 ± 68.67
Number of patients with RF positivity at low titer** (n, %)	2/16 (12.5%)
Number of patients with ACPA positivity** (n, %)	0/16 (0%)
25(OH)D (ng/mL), mean ± SD	22.68 ± 11.28
PTH (pg/mL), mean ± SD	69.62 ± 38.09
Ca (mg/dL), mean ± SD	9.34 ± 0.30
P (mg/dL), mean ± SD	3.17 ± 0.96

Supplementary Table S1. Baseline clinical, demographic, and laboratory characteristics of controls with non-inflammatory rheumatic disease

Legend with abbreviations

25(OH)D: 25-hydroxyvitamin D; **ACPA**: Anti-citrullinated protein antibodies; **BMI**: Body Mass Index; **Ca**: Calcium; **CRP**: C-reactive protein; **DOAC**: Direct oral anticoagulant; **ESR**: Erythrocyte Sedimentation Rate; **IQR**: Interquartile range; **NSAIDs**: Nonsteroidal anti-inflammatory drugs; **P**:

Phosphorus; **PLT**: Platelets; **PPIs**: Proton pump inhibitors; **PTH**: Parathyroid hormone; **RF**: Rheumatoid factor; **SD**: Standard deviation; **V0**: Visit at time 0;

* Mechanical pain was defined as having these features: worsening with activity or use of the joint, improvement with rest, minimal morning stiffness (typically less than 30 minutes)

* Considering the retrospective nature of the study, RF and ACPA for hand OA patients were available only for a subgroup of patients in whom differential diagnosis with RA was needed. In patients without these values, there was no clinical suspicion for concomitant inflammatory rheumatic diseases.

**Outcome**	**Baseline unadjusted 25OHD concentrations**	**p-value**	**Baseline adjusted 25OH concentrations for the baseline prednisone dose**	**p-value**
Total number of PMR relapses	β = 0.0422	0.214	β = 0.0427	0.214
Cumulative prednisone dosage	β = 73.1	0.320	β = 73.1	0.320
Prednisone discontinuation	OR = 1.060	0.38	OR = 1.055	0.38
DMARD requirement	OR = 0.996	0.946	OR = 0.996	0.93

Supplementary Table S2. Influence of baseline serum 25(OH)D concentrations on long-term clinical outcomes. The table displays the results of univariate and multivariate regression analyses. The multivariate models are adjusted for baseline prednisone dose to assess the relationship between vitamin D levels and key outcomes, including relapses, cumulative prednisone dose, discontinuation rates, and DMARD requirement

Abbreviations: 25(OH)D, 25-hydroxyvitamin D; β, beta coefficient; DMARD, disease-modifying anti-rheumatic drug; OR, Odds Ratio; PMR, polymyalgia rheumatica

Baseline feature	Patients achieving remission at third month (n = 8)	Patients with persistent active disease at third month (n = 6)	p-value
Age (years)	75 ± 8	75 ± 8	0.93
Female sex, ratio (%)	3/8 (38%)	3/6 (50%)	0.64
BMI	25.3 ± 4.7	28.2 ± 2.7	0.26
Disease duration (days)	88 ± 57	113 ± 92	0.52
ESR (mm/h)	45 ± 22	74 ± 53	0.30
CRP (mg/L)	38 ± 29	36 ± 58	0.93
Number of comorbidities	3.8 ± 1.9	3.2 ± 2.2	0.62
Mean cumulative prednisone dosage at third month (mg)	1009 ± 513	742 ± 535	0.41

Supplementary Table S3. Differences in baseline features of patients achieving remission or persistent active disease at third month with available dosages of 25-hydroxy vitamin D serum concentrations at baseline and at third month

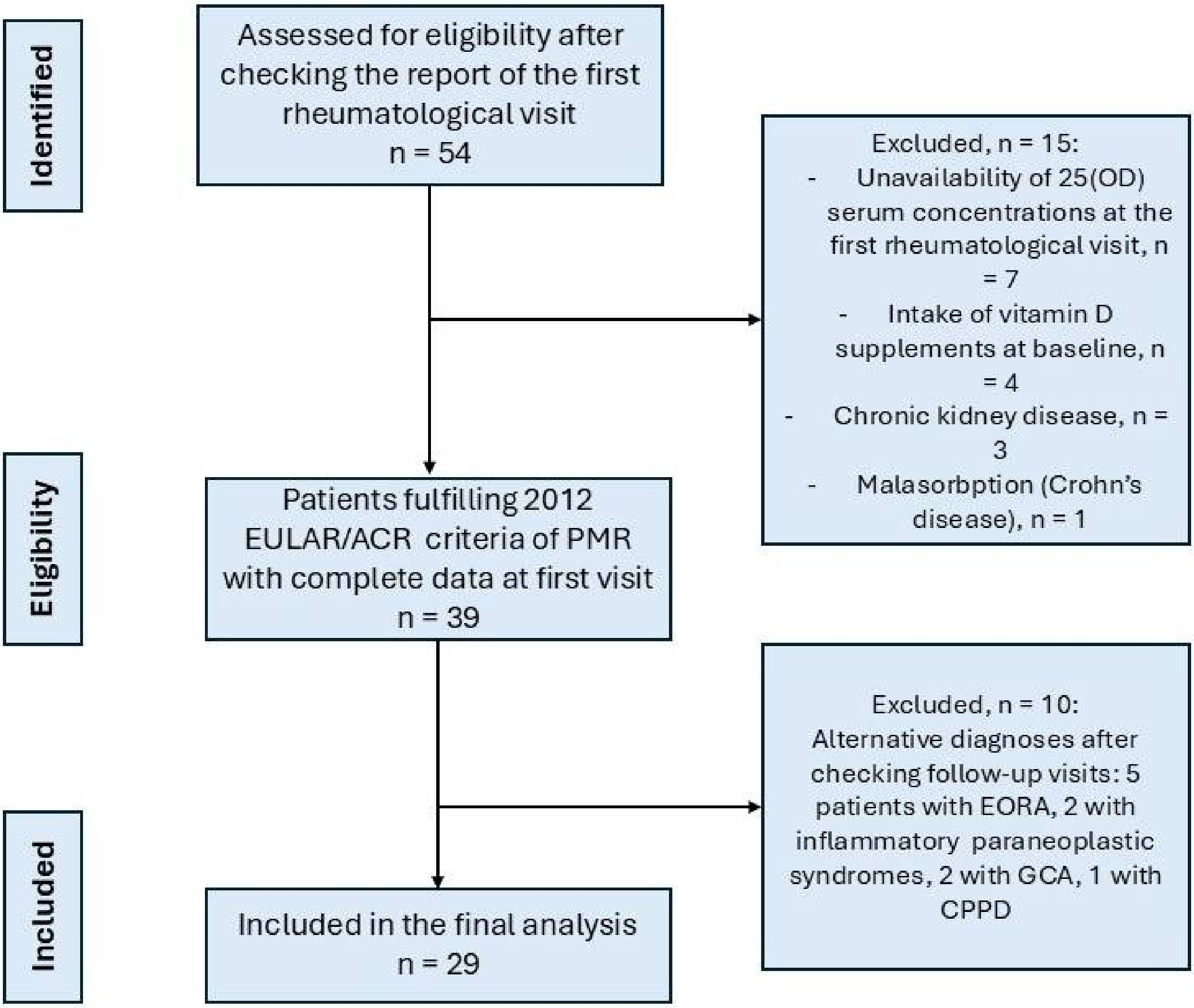

Supplementary Figure S1. Flow diagram of participant selection

Abbreviations: 25(OH)D: 25-hydroxyvitamin D; **CPPD:** calcium pyrophosphate crystal disease; **EORA:** elderly onset rheumatoid arthritis; **GCA**: giant cell arteritis; **EULAR/ACR:** European Alliance of Associations for Rheumatology/American College of Rheumatology

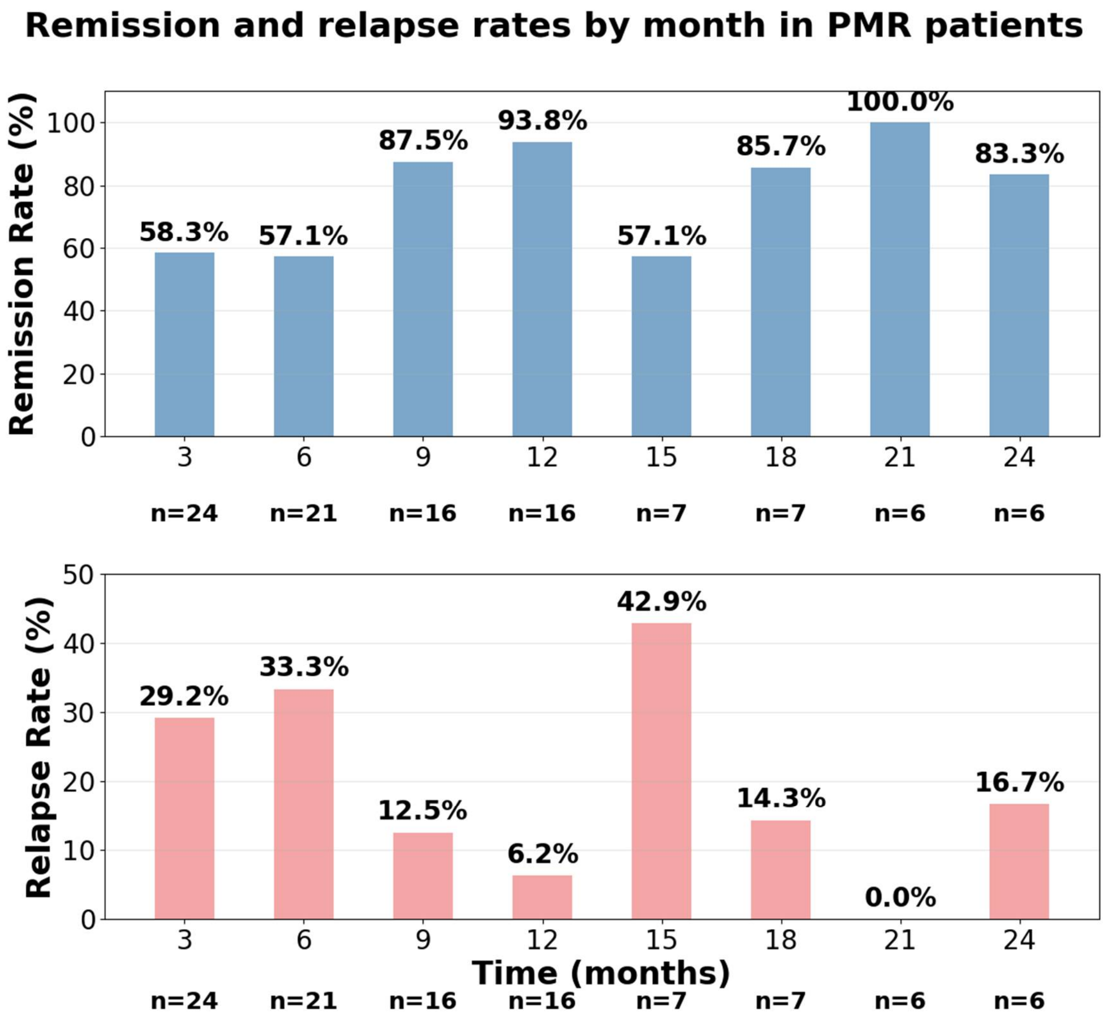

Supplementary Figure S2. Clinical outcomes in PMR patients. The histograms depict the clinical outcomes during the follow-up period, illustrating the monthly rates of patient remission (above) and disease relapse (below).
